# Characterization of recombinant OspA in two different *Borrelia* vaccines with respect to immunological response and its relationship to functional parameters

**DOI:** 10.1186/s12917-018-1625-7

**Published:** 2018-10-16

**Authors:** Deborah A Grosenbaugh, Karelle De Luca, Pierre-Yves Durand, Bradley Feilmeier, Kristopher DeWitt, Cecile Sigoillot-Claude, Marie-Line Sajous, Michael J Day, Frederic David

**Affiliations:** 1 Merial Inc., Boehringer Ingelheim, Athens, GA USA; 2 MERIAL S.A.S., Boehringer Ingelheim, Lyon, France; 3 Bristol Veterinary School, University of Bristol, Langford, UK

**Keywords:** OspA, Lyme vaccine, Borrelia

## Abstract

**Background:**

Prevention of Lyme disease in dogs in North America depends on effective vaccination against infection by the tick vector-born spirochete *Borrelia burgdorferi*. Most vaccines effectively prevent spirochete transmission to dogs during tick feeding based on immunization with the outer-surface lipoprotein A (OspA) of *B. burgdorferi*. More recently, vaccines containing additional OspC protein moieties have been introduced. These are designed to enhance protection by forming a second line of defense within the vertebrate host, where OspC expression replaces OspA as the dominant surface antigen. However, supportive data for demonstration of OspC mediated protection is still lacking. Since OspA immunogenicity is of paramount importance to protection against spirochete transmission; this study was designed to compare the immunogenicity of two commercially available vaccines against the *Borrelia burgdorferi* OspA. We further characterized OspA antigen fractions of these vaccines with respect to their biochemical and biophysical properties.

**Results:**

Two groups of beagle dogs (*n* = 9) were administered either: (1) a nonadjuvanted/monovalent, recombinant OspA vaccine (Recombitek® Lyme) or (2) an adjuvanted, recombinant OspA /OspC chimeric fusion vaccine (Vanguard® crLyme). The onset of the anti-OspA antibody response elicited by the nonadjuvanted/monovalent OspA vaccine was significantly earlier than that for the bivalent OspA /OspC vaccine and serum borreliacidal activity was significantly greater at all post-vaccination time points. As expected, only dogs inoculated with the bivalent OspA/OspC vaccine mounted a humoral anti-OspC response. However, only three out of nine dogs in that group had a positive response. Comparison of the OspA vaccine structures revealed that the OspA in the nonadjuvanted/monovalent vaccine was primarily in the lipidated form, eluting (SEC-HPLC) at a high molecular weight, suggestive of micelle formation. Conversely, the OspA moiety of the OspA/OspC vaccine was found to be nonlipidated and eluted as the monomeric protein.

**Conclusions:**

We hypothesize that these structural differences may account for the superior immunogenicity of the nonadjuvanted monovalent recombinant OspA vaccine in dogs over the adjuvanted OspA fraction of the OspA/OspC vaccine.

## Background

Canine Lyme disease is caused by the spirochete *Borrelia burgdorferi*, which is transmitted by *Ixodes spp.* ticks. Prevention of disease in dogs is based on tick control and effective vaccination. There are several vaccines on the market. Most of them effectively prevent spirochete transmission to dogs during tick feeding based on immunization with the outer-surface lipoprotein A (OspA) of *B. burgdorferi* [1, 2]. OspA has been shown to mediate humoral-mediated immunity in dogs [3] and cell-mediated immunity in humans and mice [4]. OspA is a major surface antigen of the *B. burgdorferi* spirochete, expressed during midgut colonization of its arthropod vector, and is downregulated when the bacterium enters the vertebrate host [5]. Therefore, the mammalian host antibody response can function as the first line of immunological defense against transmission of disease by neutralizing the organism within the tick vector after attachment and intake of host blood; thereby interfering with transmission.

More recently, vaccines containing OspC protein moieties have been investigated [6–8]. These are designed to enhance protection by forming a second line of defense within the vertebrate host, where OspC expression replaces OspA as the dominant surface antigen and results in a shift to anti OspC antibody production. OspC cell-mediate immunity has been demonstrated in acutely infected humans [9] and may also play and role in canines.

However, supportive data for demonstration of OspC mediated protection is still lacking. Immunization with OspC alone failed to protect mice from challenge by either *B. burgdorferi* isolates or infected wild-tick challenge [10]. Interestingly, canine vaccine efficacy studies only consider protection from challenge in the context of OspC and OspA antigen co-administration [6, 7]. Moreover, the variability among OspC genotypes within the *Borrelia burgdorferi* sensu stricto provides a challenge for the production of a broadly protective OspC-based vaccine [11]. Thus it is critical that the OspA component of either a monovalent OspA or a multivalent OspA/OspC vaccine elicits an immunological response that affords robust protection from spirochete transmission during tick feeding.

Since OspA immunogenicity is of paramount importance to protection against spirochete transmission, we compared immunological response to OspA of two commercially available vaccines: a nonadjuvanted/monovalent, recombinant OspA vaccine (Recombitek® Lyme, Merial, Inc.) and an alum-adjuvanted, recombinant OspA, chimeric OspC vaccine (VANGUARD® crLyme, Zoetis). We followed serological responses to-OspA, OspC as well as borreliacidal activity. Surprisingly, it appeared that the nonadjuvanted OspA elicited a more robust immunological response than the adjuvanted vaccine. In an attempt to understand these results, we further characterized OspA antigen fractions of these vaccines with respect to their biochemical and biophysical properties. It is concluded that the nature of the OspA antigen may dramatically impact its immunogenicity which must be considered in vaccine development.

## Methods

### Animals

Twenty-one, purpose-bred, male (*n* = 11) and female (*n* = 10) beagle dogs (Marshall BioResources, North Rose, NY 14516) were used in the study. The experimental unit was the single animal. All dogs had received routine core vaccination (canine distemper virus, canine adenovirus, canine parainfluenza virus, canine parvovirus, *Bordetella bronchiseptica*), but no previous vaccination for Lyme disease. Dogs were acclimated to the housing facility and handling procedures prior to entering the study. Dogs were 3 to 4 months of age at first vaccination and weighed between 3.5 and 4.5 kg. All were deemed clinically healthy based on physical examination. All animals were observed daily throughout the study to monitor clinical condition. Upon conclusion of the study, animals were returned to Animal Resources for assignment to other studies.

Dogs were housed separately by sex and weight (3 to 4 dogs/run), and commingled with respect to vaccination status in conventional housing. Dogs were fed an appropriate commercially available adult dog food and water was provided ad libitum. Housing, animal care, and study protocol were approved by the institutional ethics committee (Merial Institutional Animal Care and Use Committee.

### Blinding and randomization

Animals were randomized into one of two treatment groups and negative controls using littermates as a factor; and were housed in separate runs by gender, commingled and randomly assigned to pens using treatment as a factor. The random allocation sequence listing was generated by a statistician using SAS v9.4 (SAS Institute, Cary, NC). Treatments were administered in accordance with a randomization table.

All personnel performing clinical assessments, sample collection and laboratory analyses were blinded to the animal/treatment group assignments.

### Vaccination

Dogs were vaccinated with one of two recombinant vaccines.

- A nonadjuvanted, purified *B. burgdorferi* outer surface protein A (OspA) antigen derived from a bacterial recombinant vector. The OspA antigen corresponds to the active ingredient of Recombitek® Lyme (Merial, Inc.) and was formulated at the commercial release dose (*n* = 9).
- An alum-adjuvanted, recombinant OspA, chimeric outer surface protein (OspC) consisting of seven OspC subtypes. VANGUARD® crLyme (Zoetis) at the commercial dose (*n* = 9).
- Negative control animals remained unvaccinated (*n* = 3).

Both vaccines were administered in a volume of 1 ml, by subcutaneous injection, in the right and left flank, on Days 0 and 21, respectively, followed by a 5-month booster.

### Sample collection

Approximately 10 mL of blood were collected prior to the first and second vaccination, and at 14, 21, 28, 56, 64, 112 days following the second vaccination, and again at 14 days following the 5-month booster. Blood was collected into serum-separator tubes from the jugular vein of unsedated dogs using a 21 g ¾″ Surflo® butterfly catheter (Terumo, Tokyo, Japan). Serum was separated by centrifugation, and stored frozen at − 20 °C.

### Serological response

All serum samples were assayed using a fluorescent bead-based multiplex assay for the simultaneous detection of *B. burgdorferi* OspA, OspC, and OspF [12]. These tests were performed by Cornell University Veterinary Diagnostic Laboratory, Ithaca NY.

### Serum borreliacidal activity

A modified Borreliacidal Assay was developed and performed based on literature references [13–15]. Briefly, *B. burgdorferi* cultures were maintained in BSK-H Media (Sigma, Ref# B8291-500 mL) at 33 °C under static growth conditions. *B. burgdorferi* cultures were quantified using a Petroff-Hausser counting chamber under dark field microscopy and diluted to a target of 1 × 10^7^ cells/mL in fresh media.

The canine serum samples were filter sterilized using a 0.2 μm membrane prior to use, then heat inactivated at 56 °C for 10 min to prevent endogenous complement activity. Each serum sample was diluted two-fold 14 times (1:2 to 1:16,384) by adding 0.2 mL sera to 0.2 mL fresh BSK-H media in 2 mL cryogenic vials (Corning, Ref# 430659). Guinea pig complement (Calbiochem, MilliporeSigma, Ref# 234395-5ML) was filter-sterilized using a 0.2 μm membrane and then added to the diluted *B. burgdorferi* culture at a ratio of 6.67 mL Guinea pig complement to 100 mL *B. burgdorferi* culture.

To perform the assay, 0.2 mL of the culture-complement mixture was mixed with 0.2 mL of the serum dilutions and incubated at 33 °C for 16 h. After 16 h, an additional 0.8 mL of fresh BSK-H media was added to each vial. A culture-only control (without the addition of complement or dog serum) was set up to ensure normal bacterial growth. Additionally, a culture-complement (without the addition of dog serum) was set up to ensure that the complement was not resulting in indiscriminate *B. burgdorferi* mortality and cell lysis. All dilutions of each of the samples were read after 4 days using dark field microscopy at 400× magnification. The viable and motile *B. burgdorferi* cells were quantified visually using a Petroff-Hausser enumeration chamber. The borreliacidal serum antibody titer was determined by taking the reciprocal of the highest serum dilution needed to induce a minimum of 50% cell lysis relative to the culture complement control.

### Measurement of total OspA-specific immunoglobulin G and IgG subclasses

The concentration of OspA specific antibodies of the IgG1, IgG2, IgG3 and IgG4 subclasses and the total anti-OspA IgG antibody relative titers were measured using enzyme-linked immunosorbent assays (ELISA). ELISA techniques were based on detection of anti-OspA IgG by polyclonal rabbit anti-dog IgG antibodies or monoclonal mouse anti-dog IgG1, IgG2, IgG3 or IgG4 subclasses. Briefly, OspA antigen (MERIAL, Lyon, France), in PBS without Ca_2_^+^Mg_2_^+^, was coated onto the wells of a microtitration plate and incubated overnight at 5 °C. After five washes and a blocking step (5% BSA in PBS), serial dilutions of sera were incubated on the coated plate for 90 min at 37 °C. Biotin-conjugated anti-dog IgG (rabbit anti-dog IgG heavy and light chains [H&L]; Nordic Laboratories, Tilburg, The Netherlands) was then added. Binding of total IgG was detected with streptavidin-HRP (Jackson ImmunoResearch, Europe Ltd., United Kingdom). To detect binding of IgG subclasses, mouse anti-dog IgG1, IgG2, IgG3 or IgG4 subclass monoclonal antibodies were added at dilutions of 1/20000e, 1/70000e, and 1/3000e (IgG3 and IgG4) respectively in 0.05% PBS/Tween and 1% BSA. Subsequent binding was revealed using a peroxidase-conjugated goat anti-mouse IgG antibody (Jackson ImmunoResearch, Europe Ltd., United Kingdom) at a dilution of 1/5000e in 0.05% PBS/Tween and 1% BSA. Secondary and tertiary reagents were incubated for 90 min at 37 °C. Finally, substrate (tetramethylbenzidine) was added to each well. After an incubation period of 30 min at ambient temperature, H_2_SO_4_ 4 N solution was added as a blocking agent. The absorbance in each well was quantified by spectrophotometry. The antibody titers were calculated by regression and expressed as Log_10_OD_50_.

### SPR (surface plasma resonance) assays

SPR assays were performed on a BIAcore T200 (GE Healthcare Bio-Sciences AB, Sweden) using kits and buffers provided by the supplier according to the manufacturer’s instructions. A CM5 chip was immobilized with OspA or irrelevant proteins. MAbs or sera on test were diluted in running buffer and injected onto the chip. The resonance unit (RU) obtained with each pair of antigen/antibodies was monitored. The dissociation constants and avidity are calculated from half-lives using the BIAcore software, and expressed in seconds.

### OspA lipidation

Samples were analyzed on a Beckman System Gold HPLC (model 126, Beckman Coulter Life Sciences, Brea, CA, USA) with a TDA detector (Model 302, Viscotek, Malvern Instruments Ltd., Worcestershire, UK), using a Zorbax 300SB-C3 column (4.6 × 150 mm; 5 μm) and a Zorbax 300SB-C3 Guard 5 μm pre-column (Agilent, Santa Clara, CA, USA). Mobile phase used for gradient elution: A = 0.1% (*v*/v) TFA in water and B = 0.1% (v/v) TFA in ACN. The elution program comprised a flow rate of 1.5 mL/min with linear gradient from 5% B to 59% B over a period of 2 min, a flow rate of 1.5 mL/min at 59% B for 3 min, a flow rate of 1.5 mL/min with linear gradient from 59% B to 100% B over a period of 2 min and a flow rate of 1.5 mL/min at 100% B for 5 min. The column temperature was controlled at 40 °C. Detection was performed at UV 219 nm. Sample volume injected was 10 μl. OspA desorption from aluminum gel was performed by adding 150 mg of tri-sodium citrate per 1 ml of vaccine and incubation overnight at 5 °C under agitation. Aluminum gel was removed by centrifugation 10 min at 2190 g and supernatant was concentrated using ultrafiltration spinners 10 k MWCO according supplier recommendations (ThermoFischer, Rockford, IL, USA).

### Statistical analysis

Statistical analyses were performed using SAS v9.4 (SAS Institute, Cary, NC). The Log_10_ serum antibody responses to OspA and borreliacidal activity were compared between the groups on each day using a linear model with group, day and group by day as fixed effects. All animals had negative or equivocal serology on Day-7, so the baseline serology was not used as covariate. Data from the same animal were recognized by the model using an AR(1) correlation structure. The least squares means (LSM) of Log_10_ serum antibody responses were compared among groups by day. The *p*-values were adjusted for multiple comparisons using Tukey’s method. The central values with respect to the ELISA and SPR assays were compared among the two vaccinated groups by day using Student’s t-test when applicable, Wilcoxon non parametric test otherwise. Dispersions were compared by mean of F-tests.

## Results

### Humoral antibody response

**Fig. 1:**
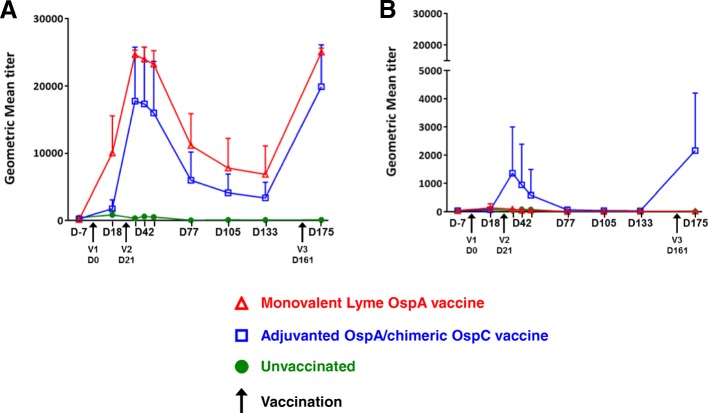
Humoral Antibody Response. Geometric mean of the borreliacidal serum antibody titers following vaccination with a recombinant, monovalent OspA vaccine and an adjuvanted, recombinant OspA, chimeric OspC vaccine as compared to unvaccinated controls. Serum samples from all dogs were assayed for serum antibody to Osp A (**a**) and Osp C (**b**)

In order to evaluate the intensity of the anti-OspA response, serum samples from dogs from both vaccination cohorts (*n* = 9) were assayed for serum antibody to Osp A using a multiplex assay (Fig. 1a). The non adjuvanted/monovalent OspA vaccine produced an early onset response by 18 days following a single vaccination, that was significantly greater than that induced by the adjuvanted OspA/chimeric OspC vaccine (*p* = 0.0008). At all other time points, the mean anti-OspA serum antibody titers in response to vaccination with the nonadjuvanted OspA vaccine was higher than that of the adjuvanted OspA/OspC vaccine but these differences were not statistically significant.

**Fig. 2:**
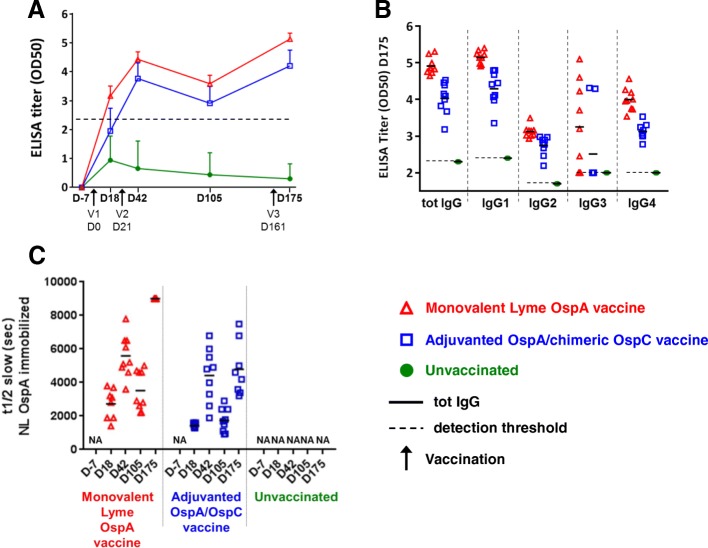
IgG-Specific Humoral Antibody Response. The total anti-OspA IgG antibody titers along the complete kinetics (**a**), Anti-OspA total IgGs and the four IgG isotypes at D175 (**b**) and antibody avidity by SPR (**c**) following vaccination with a recombinant, monovalent OspA vaccine and an adjuvanted, recombinant OspA, chimeric OspC vaccine on Day 0, Day 21, and Day 161 as compared to unvaccinated controls

Since IgG subclasses display different functions, we investigated the quality of the anti-OspA response by measuring total IgG titers, as well as IgG1, IgG2, IgG3 and IgG4. In our hands the available commercial ELISAs were not strictly specific to each of the individual IgG subclasses. Consequently, IgG subclass-specific ELISAs were developed internally from previously established methods [16] and illustrated in Fig. 2a & b. The central values of the total IgG titers were significantly higher in the dogs vaccinated with the monovalent vaccine for each time point (D18: *p* < 0.001; D42: *p* = 0.004; D105: *p* = 0.006; D175: *p* < 0.001). There was also a trend of higher dispersion of the response between dogs in the adjuvanted OspA/chimeric OspC vaccine compared to the monovalent OspA vaccine at days 18, 42 and 105, which reached significance at D175 (*p* = 0.012) (Fig. 2a). These results corroborate the multiplex serological results confirming a greater and more consistent anti-OspA humoral response between dogs with the monovalent OspA than with the bivalent vaccine, even after boosting at 5 months (*p* < 0.001) (Fig. 2b). The subclasses displayed the same chronological profiles as the total IgG, with the IgG1 response predominating over IgG2, -3 and -4 for both vaccines, as illustrated, at D175 (Fig. 2b). With the exception of IgG3, which exhibits a highly heterogeneous response, all other subclasses resulted in significantly higher titers in response to inoculation with the monovalent vaccine than with the bivalent vaccine (IgG1: *p* < 0.001; IgG2: *p* = 0.002; IgG4: *p* < 0.001), with the IgG1 response being more homogeneous between the dogs vaccinated with the monovalent vaccine (*p* = 0.012).

Antibody avidity measurements using surface plasmon resonance (SPR) (Fig. 2c) showed that vaccination with the non adjuvanted monovalent OspA resulted in the production of large amounts of specific, high-avidity antibodies, which increased after each vaccination, with all dogs consistently reaching a maximum value after the last vaccination at 5 months. In contrast, the dogs vaccinated with OspA/chimeric OspC vaccine mounted a production of specific antibodies, but with much lower avidity, which was not improved by the third vaccination at 5 months. At D18, D105 and D175, the avidity response generated in the monovalent group was significantly superior to the OspA/OspC chimeric group (D18: *p* = 0.003; D105: *p* = 0.005; D175: *p* < 0.001). The response by the unvaccinated dogs confirmed the absence of specific anti-OspA antibodies (Fig. 2c). Taken together these results demonstrate a higher avidity response elicited by the monovalent OspA compared to that of the bivalent OspA/OspC vaccine.

Since the bivalent vaccine OspA antigen relies on support by a chimeric OspC antigen as its mechanism of action, serum samples from all dogs were also assayed for serum antibody to OspC (Fig. 1b). As expected, the OspC response to the monovalent OspA vaccine was comparable to that of the unvaccinated controls. Response to vaccination to the OspA/chimeric OspC vaccine followed a temporal pattern similar to that of the OspA response, but was highly variable between individual dogs with respect to magnitude. Only three out of nine dogs exceeded the threshold for positivity 2 weeks following the second inoculation.

### Serum borreliacidal activity

**Fig. 3:**
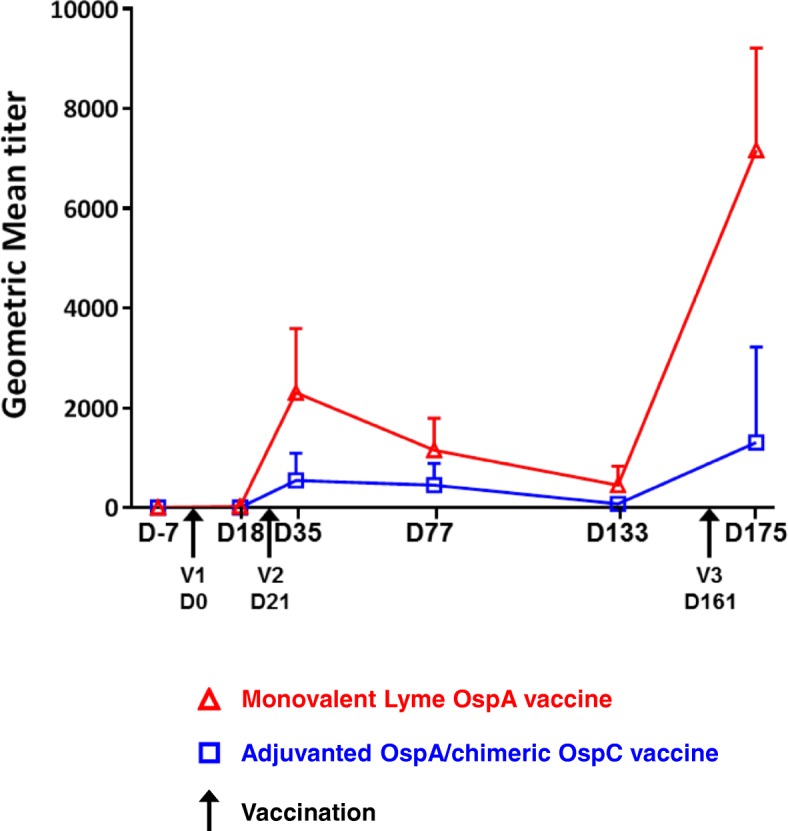
Serum Borreliacidal Activity. Geometric mean titers of borreliacidal activity following vaccination with a recombinant, monovalent OspA vaccine and an adjuvanted, recombinant OspA, chimeric OspC vaccine

The functionality of the antibody response was further explored by measuring borreliacidal activity in serum samples from all dogs at selected time points (Fig. 3). Post-vaccination borreliacidal activity of the sera from dogs receiving nonadjuvanted/monovalent OspA were consistently and significantly higher 18 days following the first vaccination (*p* = 0.0053), 14 days following the second vaccination (*p* = 0.0011) and 14 days after the 5 months booster vaccination (*p* = 0.0021) than those from dogs that received the OspA/chimeric OspC vaccine.

### OspA biochemical properties and their impact on antigen conformation

**Fig. 4:**
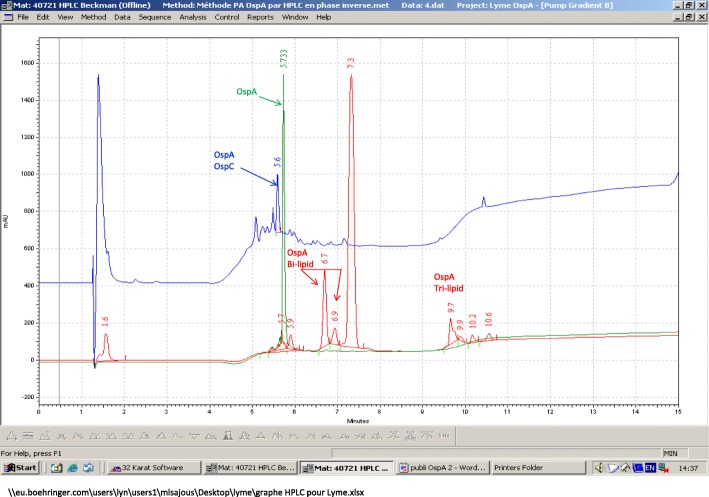
OspA biochemical properties and their impact on antigen conformation. HPLC separation (retention time) of the lipidation states of Recombitek ® Lyme and Vanguard ® crLyme compared to a nonlipidated OspA standard

To investigate possible causes for the observed difference in the serological response between the two vaccines, the lipidation state of their respective OspA active ingredients were determined by reverse-phase high performance liquid chromatography (RP-HPLC) and compared to a nonlipidated OspA mutant. The latter was created by deleting the N-terminal cystein (OspA nonlipidated control). The OspA active ingredient from the nonadjuvanted/monovalent vaccine eluted as a mix of bi- and tri-lipidated forms. It contained no measurable nonlipidated proteins, whereas the OspA active ingredient from the adjuvanted OspA/chimeric OspC vaccine matched the profile of the non lipidated control (Fig. 4). Size exclusion HPLC (SEC-HPLC) coupled to a tetra-detector array (TDA) composed of a UV-IR-LS-Viscometer indicated a molecular mass of the lipidated OspA from the nonadjuvanted/monovalent vaccine to be 418 kDa, corresponding to a diameter of 18 nm. Molecular mass of the non-lipidated OspA control was measured at 34 kDa for a diameter of 6.4 nm, which is in accordance with the predicted monomeric state.

To further investigate the nature of the size increase of the lipidated OspA active ingredient from the nonadjuvanted/monovalent vaccine, both lipidated recombinant OspA and the nonlipidated OspA control were analysed by Fourier transform infrared spectroscopy (FTIR) to assess secondary structure. Both spectra showed similar percentages of secondary structural elements of both proteins. Moreover, no intermolecular beta-sheets were detected for lipidated OspA. Thus, the lipidated proteins did not appear to form aggregation patterns, suggesting that the multimeric profile of nonadjuvanted/monovalent OspA proteins is due exclusively to the presence of the lipid moities.

## Discussion

In this study, we compared the immunogenicity of two different OspA antigens from commercially available canine vaccines: a nonadjuvanted/monovalent, recombinant OspA vaccine (Recombitek® Lyme) and an adjuvanted, recombinant OspA /OspC chimeric fusion vaccine (Vanguard® crLyme).

Our results show that the OspA monovalent, nonadjuvanted vaccine elicited an earlier onset of immunity with a significantly higher antibody response than the adjuvanted vaccine after the first vaccination. Following the second and third vaccinations the average antibody titers were consistently higher at all time points in the dogs receiving the nonadjuvanted vaccine as compared to the dogs receiving the alum-adjuvanted vaccine, although these differences were not significant. Alum is characterized as primarily potentiating a strong Th2 response [17]. It is possible that a different adjuvant, potentiating Th1 responses, may have influenced the results obtained with the recombinant OspA /OspC chimeric fusion antigen.

The humoral response was explored qualitatively by measuring IgG subclasses distribution and functionally by quantifying complement-mediated borreliacidal activity. It is shown that the IgG1 response predominates in both vaccines; but the nonadjuvanted vaccine mounted a more homogenous IgG response with antibodies of greater avidity than that elicited by the adjuvanted vaccine. In addition, the complement-mediated borreliacidal activity induced by the non adjuvanted OspA vaccine was significantly higher than that of the adjuvanted OspA/OspC vaccine at all time points.

The IgG1 subclass described for humans resembles that of the canine IgG-B counterpart [18]. In dogs, it has been identified to be involved in antibody-dependent cell-mediated cytotoxicity (ADCC), cell-mediated cytotoxicity (CDC) and in C1q complement binding. The IgG1 isotype response is pronounced in human patients recovering from subacute *Borrelia* infection and may contribute to clearing of the infection [19].

This may have implications regarding the functionality of the humoral immune response as a whole. Taken together, our results suggest that the greater levels of anti-OspA IgG antibody elicited by the non-adjuvanted OspA vaccine and the higher avidity of these antibodies may enhance the ability of this vaccine to trigger complement fixation and, in turn, borreliacidal activity.

HPLC analysis indicates that most of the OspA in the monovalent vaccine was in the form of bi- and tri-lipidated protein, while OspA derived from the adjuvanted OspA/OspC vaccine eluted as a single, nonlipidated peak. The lipidated OspA eluted as a much larger particle (18 nm) than the nonlipidated OspA (6.4 nm). This suggests that the lipidated proteins are organized as particular elements, possibly micelles, based on the tendency of lipoproteins to organize into aggregates that internalize hydrophobic moieties. It has been shown that lipidation of OspA protein is a critical determinant of immunogenicity [20] which may, in part, account for the superior immunogenic profile of the monovalent OspA. Self-assembled micellar nanoparticles appear to increase the efficiency of antigen uptake by dendritic cells [21]. Nanoparticles can also migrate to lymph nodes, and activate nodal dendritic cells, increasing the germinal B cell response [22]. We therefore hypothesize that the lipidation of the OspA antigen present in the monovalent nonadjuvanted vaccine accounts for its superior immunogenic profile over the adjuvanted, nonlipidated OspA from the bivalent vaccine.

With regards to OspC immunogenicity, as expected, the monovalent non-adjuvanted OspA vaccine did not elicit a detectable anti-OspC antibody response. In the OspA/OspC adjuvanted vaccine group, only three out of nine dogs were confirmed positive 2 weeks after the second vaccination.

Since OspC antigens are highly variable [23], this result may be a reflection of a mismatch between the OspC antigens used in the vaccine and the antibody assay [12]. Alternatively, the low rate of seroconversion against OspC elicited by the OspA/OspC vaccine could be related to the inherent variability of OspC antibody responses in dogs [24] or the inability of the OspC chimeric antigen used in the vaccine to elicit an effective antibody response against the native OspC antigen. Whether this low seroconversion to OspC is a reflection of antigenic or immunogenic variability, it illustrates the technical challenge of inducing a broadly protective and consistent immune response against OspC in dogs. Finally, with regards to the OspC immune response, it is also possible that some of the dogs that were negative in the antibody assay performed here would have been positive if tested using assays designed to measure cell mediated immune responses.

The importance of the antibody response to OspA in mediating borreliacidal activity within the tick and consequently blocking transmission to the host has been thoroughly documented [5, 25, 26]. Considering that the protective efficacy of OspC alone remains to be demonstrated [10], the earlier onset of immunity against OspA (after the first dose of vaccine) and the greater ability to generate a borreliacidal activity conferred by the lipidated monovalent OspA vaccine are expected to be an advantageous regarding efficacy. It is acknowledged that humoral mediated immune response may not fully predict vaccine efficacy; but likewise, demonstration of superior clinical efficacy using current *Borrelia* challenge models in a limited number of genetically homogenous dogs is also not likely to reflect vaccine efficacy in the field. However, obtaining this enhanced immunogenicity by expressing a lipidated OspA active ingredient without using an adjuvant is an inherent safety advantage.

## Conclusions

Our results show that the OspA monovalent, nonadjuvanted vaccine elicited an earlier onset of immunity with a significantly higher antibody response than the adjuvanted vaccine. The functionality of the antibody response was supported by a greater concurrent increase in serum borreliacidal activity from dogs that receive the nonadjuvanted/monovalent OspA vaccine over those from dogs that received the OspA/chimeric OspC vaccine. The extent of lipidation resulting in structural differences between OspA fractions of the vaccines appear to confer an immunological advantage that obviates the need for the addition of an adjuvant .

## Abbreviations

ACN: Acetonitrile
ADCC: Antibody-dependent cell-mediated cytotoxicity
AR: Autoregression
BSA: Bovine serum albumin
CDC: Cell-mediated cytotoxicity
D: Day
ELISA: Enzyme-linked immunosorbent assay
FTIR: Fourier transform infrared spectroscopy
LSM: Least squares mean
MAbs: Monoclonal antibodies
MWCO: Molecular weight cutoff
OD_50_: Optical density 50%
OspA: Outer-surface lipoprotein A
OspC: Outer-surface lipoprotein C
PBS: Phosphate-buffered saline
RP-HPLC: Reverse phase high performance liquid chromatography
RU: Resonance unit
SEC-HPLC: Size exclusion high performance liquid chromatography
SPR: Surface plasmon resonance
TDA: Tetra-detector array
TFA: Trifluroacetic acid
